# Supplementary material for: Lactobacillus crispatus S-layer proteins modulate innate immune response and inflammation in the lower female reproductive tract
Source: Nat Commun. 2024 Dec 30;15:10879. doi: 10.1038/s41467-024-55233-7 (PMC11685708; doi:10.1038/s41467-024-55233-7)
Supplement: Supplementary file 1 — Supplementary Information [file 41467_2024_55233_MOESM1_ESM.pdf]

Supplementary Figure 1

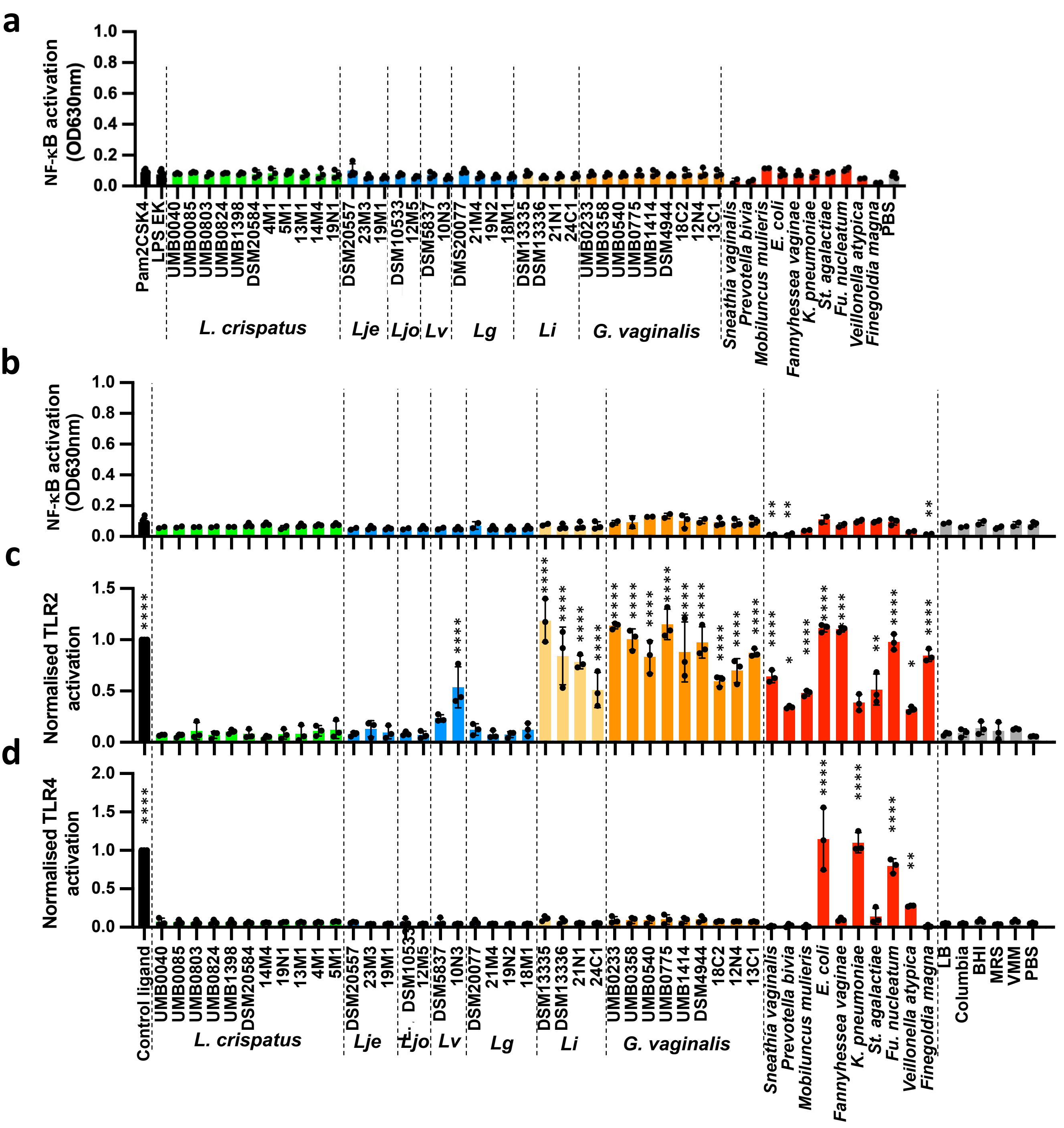

Supplementary Fig. 1: Bacteria and bacterial culture supernatant associated with preterm birth activate pro-inflammatory signaling pathways via TLR2 and TLR4. (a) The HEK Null cells were incubated overnight with the bacteria (MOI=10) and activation of the NF-κB and AP1 dependent pro-inflammatory pathways was measured. HEK Null1 parental cell line (b) HEK TLR2 (c) and HEK TLR4 (d) were incubated with 10ul of bacterial culture medium overnight. Pam2CSK4 and LPS EK were used as control ligands for HEK TLR2 and HEK TLR4 respectively. Lje: *Lactobacillus jensenii*, Ljo: *Lactobacillus johnsonii*, Lv: *Limosilactobacillus vaginalis*, *Lg: L. gasseri,* Li: *L. iners*. Data are mean +/-SD (n=3 independent experiments). One way ANOVA, Dunnett multiple comparison test against control (PBS) condition . * p<0.05, ** p<0.01, *** p<0.005, **** p<0,001.

Supplementary Figure 2

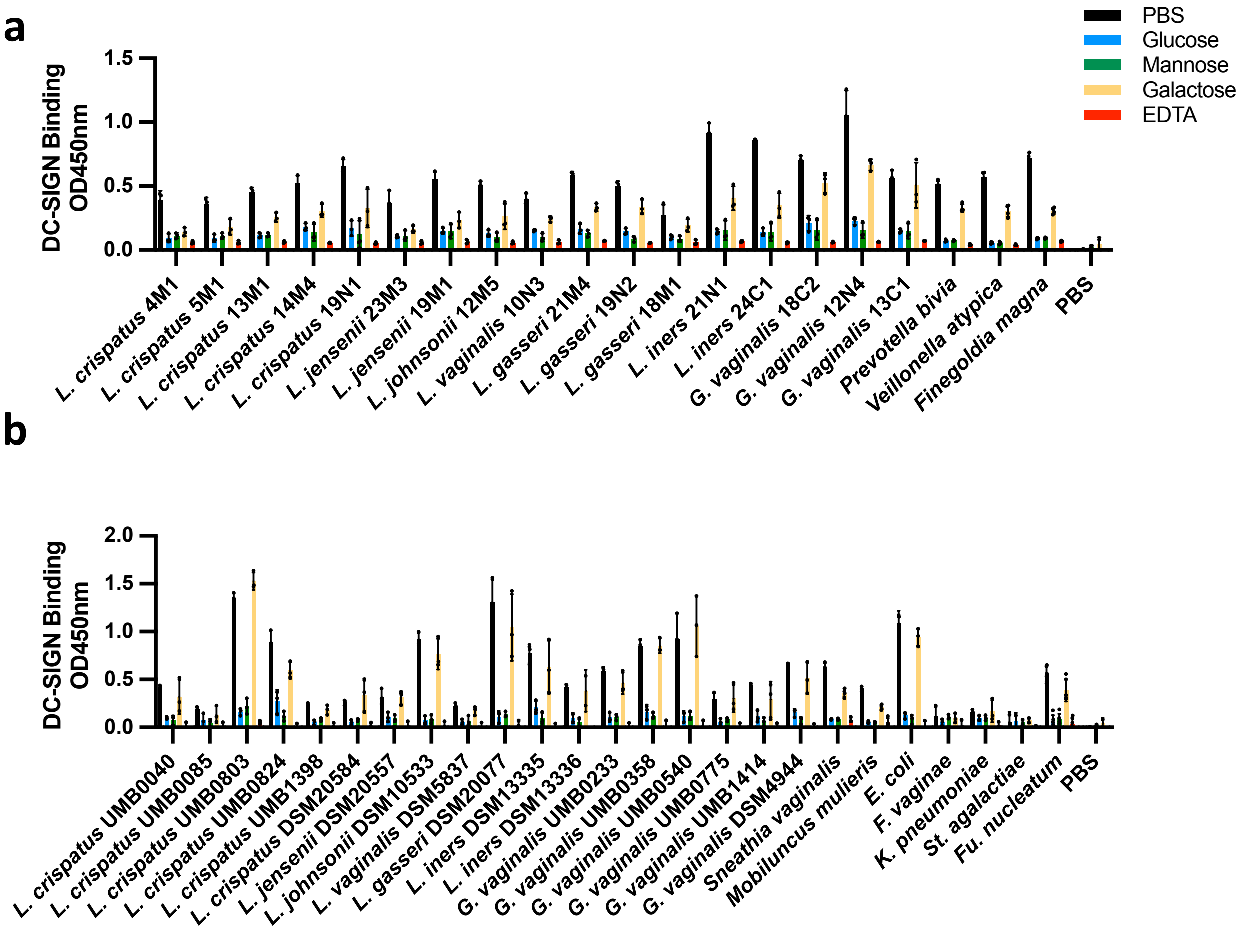

Supplementary Fig. 2: **DC-SIGN binding to the bacteria is glycan-dependent.** Bacteria isolated from vaginal swabs (a) and commercial strains and bacteria isolated from urinary tract infection patients (b) (10^6 bacteria per well) were coated in 96-well plates and tested for their capacity to bind DC-SIGN-Fc. DC-SIGN-Fc (1 µg/ml) was pre-incubated or not with 20 mM EDTA or 40 mM glucose, mannose and galactose and allowed to react with the bacteria for 2 h at RT. Bound proteins were detected using a biotin-conjugated anti-IgG Fc specific antibody and avidin-HRP and reading O.D. at 450 nm. Data are mean +/-SD (n=3 independent experiments).

Supplementary Figure 3

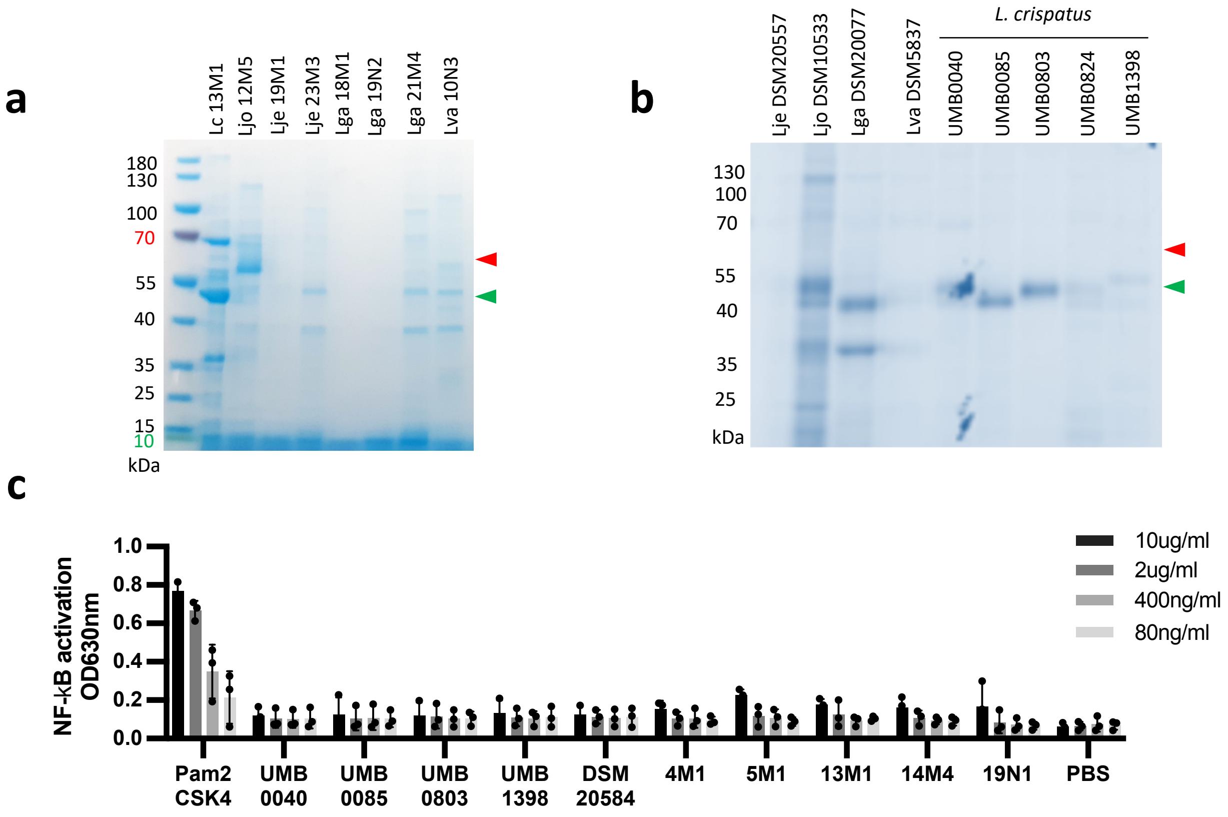

Supplementary Fig. 3: **SLPs isolated from *L. crispatus* don't activate TLR2 signaling.** (a) SDS-PAGE gel of crude SLPs extracted with LiCl 5M from vaginal lactobacilli isolates. 10ug total proteins were loaded per lane. Repersentative of three independent experiments. (b) SDS-PAGE gel of crude SLPs extracted with LiCl 5M from commercial and urine lactobacilli isolates. 10ug total proteins were loaded per lane. Repersentative of three independent experiments. (c) HEK-TLR2 cells were stimulated for 16 h with purified SLPs from the *L. crispatus* isolates and NF-κB activation was determined by measuring alkaline phosphatase activity and reading O.D. at 630 nm. Data are mean +/-SD (n=3 independent experiments).

Supplementary Figure 4

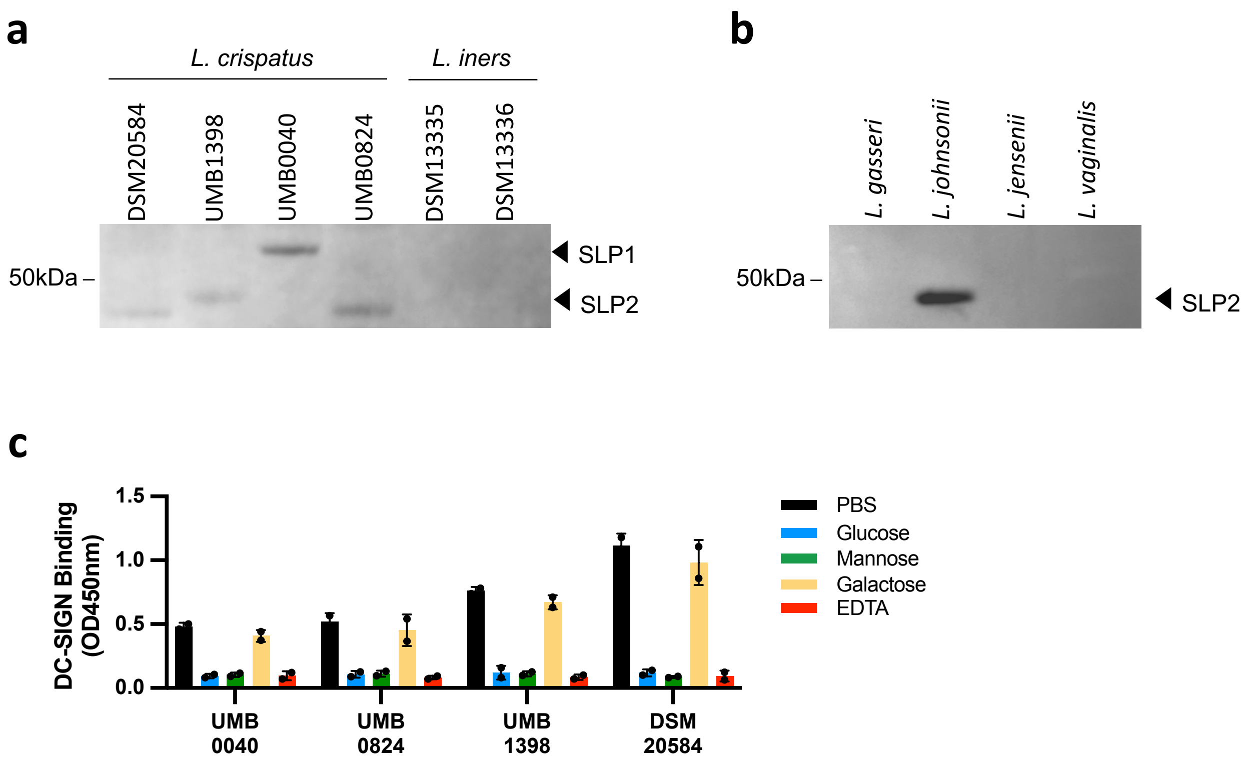

Supplementary Fig. 4: **SLPs are ligands of DC-SIGN**. (a) Western blotting results for DC-SIGN-Fc binding to LiCl 5M extracts from *L. crispatus* and *L. iners* strains (one representative of three replicates), (b) Western blotting results for DC-SIGN-Fc binding to LiCl 5M extracts from *L. gasseri* DSM20077, *L. johnsonii* DSM10533, *L. jensenii* DSM20557 and *L. vaginalis* DSM5487 (one representative of three replicates), (c) Purified SLPs isolated from *L. crispatus* strains (1ug per well) were coated in 96-well plates and tested for their capacity to bind DC-SIGN-Fc. DC-SIGN-Fc (1 µg/ml) was pre-incubated or not with 20 mM EDTA or 40 mM glucose, mannose and galactose and allowed to react with the SLPs for 2 h at RT. Bound proteins were detected using a biotin-conjugated anti-IgG Fc specific antibody and avidin-HRP and reading O.D. at 450 nm. Data are mean +/-SD (n=2 independent experiments).

Supplementary Figure 5

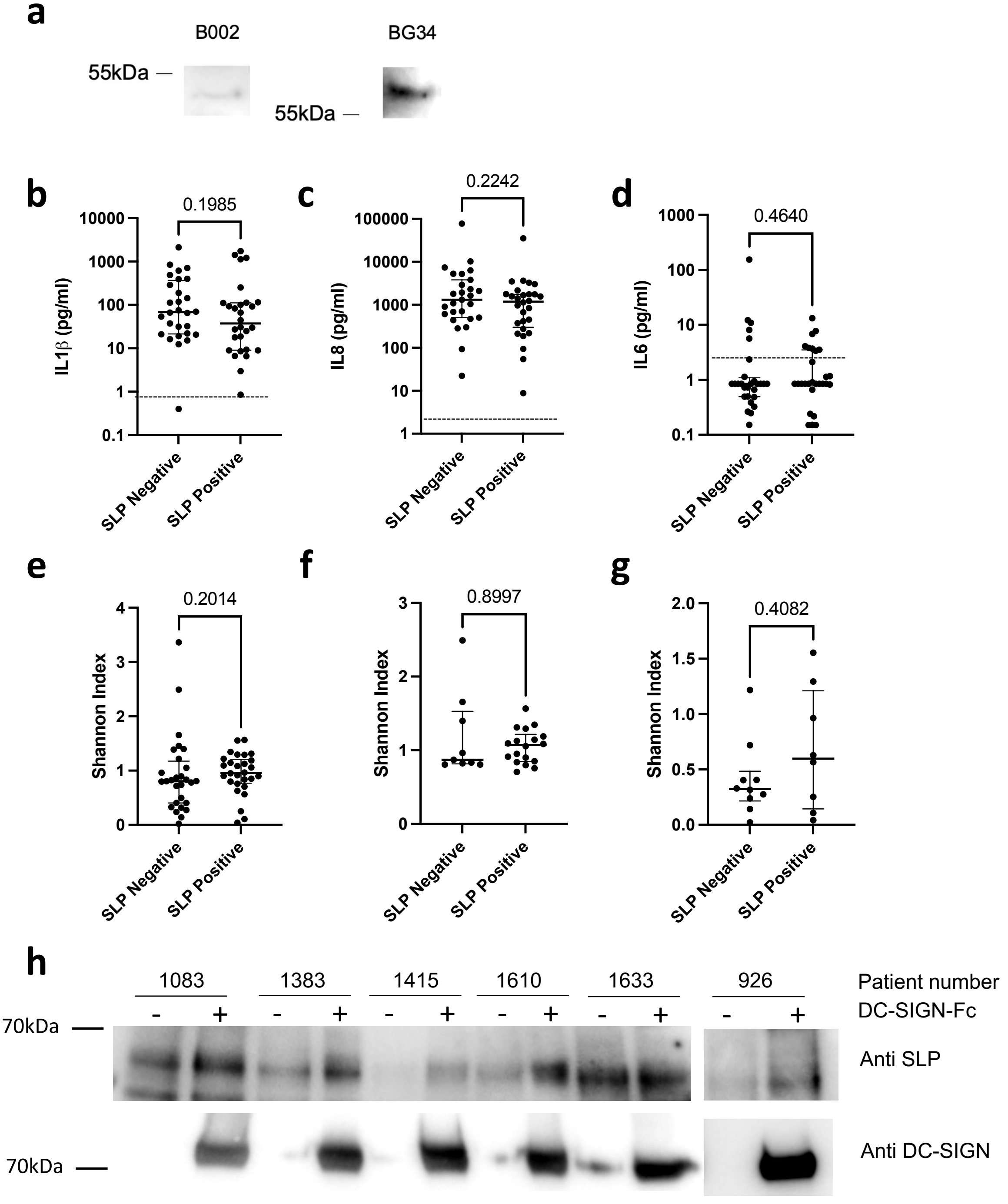

Supplementary Fig. 5: **SLPs can be detected in CVF samples**. (a) Western blotting results for anti-Surface Layer Protein antibody binding to purified CST-IV dominated cervico-vaginal fluid samples (B002 containing *L. gasseri* and BG34 containing *L. crispatus*), (b, c, d) Cervicovaginal concentrations of IL-1β (b), IL-8 (c), IL-6 (d) are shown from all the samples samples (n=56 women). Statistical analysis was performed using a one-sided Mann–Whitney test. Data are presented as median values and interquartile ranges (25th and 75th percentiles). Limit of detection is presented as dotted line. (e, f, g) Shannon diversity index of the vaginal microbiome comparing the SLP positive and negative samples across all samples (e), *L. crispatus* dominated (f) and *L. iners* dominated samples (g). Statistical analysis was performed using a one-sided Mann–Whitney test. Data are presented as median values and interquartile ranges (25th and 75th percentiles). (h) Immunoprecipitation results for DC-SIGN-Fc binding to SLPs in CVF samples. One representative if three independent experiments. Source Data are provided as a Source Data File.
