# Supplementary material for: Lactobacillus crispatus S-layer proteins modulate innate immune response and inflammation in the lower female reproductive tract
Source: Nat Commun. 2024 Dec 30;15:10879. doi: 10.1038/s41467-024-55233-7 (PMC11685708; doi:10.1038/s41467-024-55233-7)
Supplement: Supplementary file 2 — Description of Additional Supplementary files [file 41467_2024_55233_MOESM2_ESM.docx]

**Supplementary Data 1 :** Bacterial composition of the cervico-vaginal fluid samples determined by 16S rRNA gene sequencing

File name: Supplementary Data 1

Description: Table describing the bacterial composition of the cervico-vaginal fluid samples determined by 16S rRNA gene sequencing, the community state type of the sample and the presence of surface layer proteins detected by western blot in the cervico-vaginal fluids.

**Supplementary Data 2** : Commercial and UTI bacterial strains

File name: Supplementary Data 2

Description: Table describing the source of the commercial and UTI patients bacterial strains and their culture conditions.

**Supplementary Data 3**: Vaginal bacterial strains

File name: Supplementary Data 3

Description: Table describing the source of vaginal bacterial strains, details of the patients (blood group, maternal age, BMI, parity and gravida) and the bacterial culture conditions.
