# Supplementary material for: Lactobacillus crispatus S-layer proteins modulate innate immune response and inflammation in the lower female reproductive tract
Source: Nat Commun. 2024 Dec 30;15:10879. doi: 10.1038/s41467-024-55233-7 (PMC11685708; doi:10.1038/s41467-024-55233-7)
Supplement: Supplementary file 6 — Reporting Summary [file 41467_2024_55233_MOESM6_ESM.pdf]

nature portfolio

Corresponding author(s): Alexiane Decout

Last updated by author(s): Nov 14, 2024

Reporting Summary

Nature Portfolio wishes to improve the reproducibility of the work that we publish. This form provides structure for consistency and transparency in reporting. For further information on Nature Portfolio policies, see our Editorial Policies and the Editorial Policy Checklist.

Statistics

For all statistical analyses, confirm that the following items are present in the figure legend, table legend, main text, or Methods section.

n/a	Confirmed	
☐	☒	The exact sample size (*n*) for each experimental group/condition, given as a discrete number and unit of measurement
☐	☒	A statement on whether measurements were taken from distinct samples or whether the same sample was measured repeatedly
☐	☒	The statistical test(s) used AND whether they are one- or two-sided<br>*Only common tests should be described solely by name; describe more complex techniques in the Methods section.*
☒	☐	A description of all covariates tested
☒	☐	A description of any assumptions or corrections, such as tests of normality and adjustment for multiple comparisons
☐	☒	A full description of the statistical parameters including central tendency (e.g. means) or other basic estimates (e.g. regression coefficient) AND variation (e.g. standard deviation) or associated estimates of uncertainty (e.g. confidence intervals)
☐	☒	For null hypothesis testing, the test statistic (e.g. *F*, *t*, *r*) with confidence intervals, effect sizes, degrees of freedom and *P* value noted<br>*Give P values as exact values whenever suitable.*
☒	☐	For Bayesian analysis, information on the choice of priors and Markov chain Monte Carlo settings
☒	☐	For hierarchical and complex designs, identification of the appropriate level for tests and full reporting of outcomes
☒	☐	Estimates of effect sizes (e.g. Cohen's *d*, Pearson's *r*), indicating how they were calculated

Our web collection on statistics for biologists contains articles on many of the points above.

Software and code

Policy information about availability of computer code

Data collection	ELISA and QuantiBlue were acquired using Magellan (Tecan).<br>Images of the western blots and SDS-PAGE gels were recorded using the ChemiImager LAS4000 software (GE Healthcare)<br>Luminex data was acquired with MagpixTM.
Data analysis	Prism software (Graphpad Software, version 10.1.1) was used to perform statistical tests and to generate graphs.<br>Images of the western blots and SDS-PAGE gels were analysed using the ChemiImager LAS4000 software (GE Healthcare, version 1.3)<br>Luminex Data were analysed with Magpix' software package control xPONENT® (version 4.3).<br>QIIME2 bioinformatics pipeline (version 2002.2.1) and DADA2 (version 2022.2.0) were used for microbiome analyses. Shannon diversity was computed using the phyloseq package (version 1.32.0) using R (version 4.0.0).

For manuscripts utilizing custom algorithms or software that are central to the research but not yet described in published literature, software must be made available to editors and reviewers. We strongly encourage code deposition in a community repository (e.g. GitHub). See the Nature Portfolio guidelines for submitting code & software for further information.

Data

Policy information about availability of data

All manuscripts must include a data availability statement. This statement should provide the following information, where applicable:

- Accession codes, unique identifiers, or web links for publicly available datasets
- A description of any restrictions on data availability
- For clinical datasets or third party data, please ensure that the statement adheres to our policy

The data that support the findings are available in the source file.
The 16S rRNA gene sequencing data generated in this study have been deposited in the European Nucleotide Archive under the study accession code PRJEB83084. The samples accession numbers are: ERS22541924, ERS22541925, ERS22541926, ERS22541927, ERS22541928, ERS22541929, ERS22541930, ERS22541931, ERS22541932, ERS22541933, ERS22541934, ERS22541935, ERS22541936, ERS22541937, ERS22541938, ERS22541939, ERS22541940, ERS22541941, ERS22541942, ERS22541943, ERS22541944, ERS22541945, ERS22541946, ERS22541947, ERS22541948, ERS22541949, ERS22541950, ERS22541951, ERS22541952, ERS22541953, ERS22541954, ERS22541955, ERS22541956, ERS22541957, ERS22541958, ERS22541959, ERS22541960, ERS22541961, ERS22541962, ERS22541963, ERS22541964, ERS22541965, ERS22541966, ERS22541967, ERS22541968, ERS22541969, ERS22541970, ERS22541971, ERS22541972, ERS22541973, ERS22541974, ERS22541975, ERS22541976, ERS22541977, ERS22541978, ERS22541979

Research involving human participants, their data, or biological material

Policy information about studies with human participants or human data. See also policy information about sex, gender (identity/presentation), and sexual orientation and race, ethnicity and racism.

Reporting on sex and gender	This study focused on the vaginal microbiome. All the participants were of female sex.
Reporting on race, ethnicity, or other socially relevant groupings	Self reported ethnicity was recorded at the time of the visit and is included in Extended data Table 3.
Population characteristics	Pregnant women who were identified as being at risk of preterm labour were recruited from the Preterm Birth Prevention and Surveillance clinic, or prior to receiving an emergency cervical cerclage. Pregnant women are referred to the clinic at the time of their booking appointment by 12 weeks if they have a history of previous preterm birth (<34 weeks), previous preterm prelabour rupture of membranes (<34 weeks), previous mid-trimester loss (16-23 weeks), a history of cervical trauma (either cervical treatment including surgery such as previous cone biopsy and large loop excision of the transformation zone) or a uterine anomaly. Women at intermediate risk (history of cervical cerclage in a previous pregnancy or previous fully dilated caesarean section) were also seen in clinic. Women were also referred from routine antenatal care if a short cervix and/or bulging membranes is detected at the time of the routine anomaly scan (18-22 weeks). All patients provided written informed consent to donate specimens.
Recruitment	Pregnant women at high risk of preterm birth were recruited from Imperial College Healthcare NHS Trust maternity hospitals: Queen Charlotte's and Chelsea Hospital (QCCH). Patients were recruited and samples collected from June 2018 to June 2022. Exclusion criteria included: sexual activity within 72 hours of sampling, vaginal bleeding in the preceding week, HIV positive women, hepatitis B positive serology, and women under the age 18.
Ethics oversight	The study was performed under the Ethics approval REC 14/LO/0328 as part of the Vaginal Microbiome and Metabonome in Pregnancy (VMET 2) Research Study and approved by the NHS Health Research Authority (London -Stanmore Research Ethics Committee)

Note that full information on the approval of the study protocol must also be provided in the manuscript.

Field-specific reporting

Please select the one below that is the best fit for your research. If you are not sure, read the appropriate sections before making your selection.

☒ Life sciences ☐ Behavioural & social sciences ☐ Ecological, evolutionary & environmental sciences

For a reference copy of the document with all sections, see nature.com/documents/nr-reporting-summary-flat.pdf

Life sciences study design

All studies must disclose on these points even when the disclosure is negative.

Sample size	No statistical methods were used to predetermine sample sizes
Data exclusions	No data were excluded
Replication	All experiments were performed with at least 3 biological replicates to ensure reproducibility across experiments
Randomization	Randomization is not relevant to our study design.

Blinding | Blinding is not relevant to our study design. Our data don’t rely on subjective evaluation but on quantifiable measurements.

Reporting for specific materials, systems and methods

We require information from authors about some types of materials, experimental systems and methods used in many studies. Here, indicate whether each material, system or method listed is relevant to your study. If you are not sure if a list item applies to your research, read the appropriate section before selecting a response.

Materials & experimental systems

n/a	Involved in the study	
☐	☒	Antibodies
☐	☒	Eukaryotic cell lines
☒	☐	Palaeontology and archaeology
☒	☐	Animals and other organisms
☐	☒	Clinical data
☒	☐	Dual use research of concern
☒	☐	Plants

Methods

n/a	Involved in the study	
☒	☐	ChIP-seq
☒	☐	Flow cytometry
☒	☐	MRI-based neuroimaging

Antibodies

Antibodies used | Anti-hTLR1-IgG (InvivoGen, clone H2G2, mabg-htlr1-2), Anti-hTLR6-IgG (InvivoGen, clone C5C8, mabg-htlr6-2), Human TLR2 MAb (Biotechne, Clone # 383936, MAB2616), anti-DC-SIGN (R&D Systems, MAB161-100), Mouse IgG2b Isotype Control (Thermo Fisher Scientific, clone eBMG2b, 14-4732-85, lot 2288614), Mouse Control IgG1 (InvivoGen, clone T8E5, mabg1-ctrlm), Goat anti-Human IgG Fc Secondary Antibody, HRP (Thermo Fisher Scientific, A18817), Surface Layer Protein Polyclonal Antibody (Bioss, polyclonal, BS-3797), Goat Anti-Rabbit Immunoglobulins/HRP (Dako, P0448), anti-mouse-IgG-HRP (Biotechne, NBP1-75130), anti-DC-SIGN (R&D Systems, Clone # 120507, MAB161-100)

Validation |
https://www.invivogen.com/anti-htlr1-igg#specifications
https://www.invivogen.com/anti-htlr6-igg
https://www.rndsystems.com/products/human-tlr2-antibody-383936_mab2616
https://www.thermofisher.com/antibody/product/Mouse-IgG2b-kappa-clone-eBMG2b-Isotype-Control/14-4732-82
https://www.invivogen.com/control-igg1
https://www.rndsystems.com/products/human-dc-sign-cd209-antibody-120507_mab161
https://www.thermofisher.com/antibody/product/Goat-anti-Human-IgG-Fc-Cross-Adsorbed-Secondary-Antibody-Polyclonal/31413?ef_id=Cj0KCQiAzoeuBhDqARIsAMdH14EW9PSITsnJH8etQboQChJyPXYIuwU5eVILadF789duUuaqPefrqr0aAtH3EALw_wcB:G:s&s_kwcid=AL!3652!3!516608152296!!!g!!!12825517856!122158234755&cid=bid_pca_aus_r01_co_cp1359_pjt0000_bid00000_0se_gaw_dy_pur_con&gad_source=1&gclid=Cj0KCQiAzoeuBhDqARIsAMdH14EW9PSITsnJH8etQboQChJyPXYIuwU5eVILadF789duUuaqPefrqr0aAtH3EALw_wcB
https://www.biossantibodies.com/datasheets/bs-3797R
https://www.agilent.com/en/product/specific-proteins/elisa-kits-accessories/goat-anti-rabbit-immunoglobulins-hrp-affinity-isolated-2717113
https://www.bio-techne.com/p/secondary-antibodies/igg-h-l-antibody_nbp1-75130
https://www.rndsystems.com/products/human-dc-sign-cd209-antibody-120507_mab161

Eukaryotic cell lines

Policy information about cell lines and Sex and Gender in Research

Cell line source(s) | American Type Culture Collection (ATCC): VK2 E6/E7 cell line (CRL-2616)
InvivoGen: HEK-Blue Null1 (hkb-null1), HEK-Blue hTLR2 (hkb-tlr2), HEK-Blue hTLR4 (hkb-htlr4)

Authentication | Cells were purchased from authenticated suppliers.

Mycoplasma contamination | All cells were tested negative for mycoplasma.

Commonly misidentified lines (See ICLAC register) | No commonly misidentified cell lines were used in this study

Clinical data

Policy information about clinical studies

All manuscripts should comply with the ICMJE guidelines for publication of clinical research and a completed CONSORT checklist must be included with all submissions.

Clinical trial registration	REC 14/LO/0328
Study protocol	https://www.hra.nhs.uk/planning-and-improving-research/application-summaries/research-summaries/vmet-2/
Data collection	Pregnant women at high risk of preterm birth were recruited from Imperial College Healthcare NHS Trust maternity hospitals: St Mary's Hospital (SMH), Queen Charlotte's and Chelsea Hospital (QCCH) and Chelsea and Westminster Hospital (CWH); as well as from University College London Hospital (UCLH). Patients were recruited and samples collected from June 2018 to June 2022
Outcomes	Whole genomic DNA extracted from each swab was sequenced for the V1-V2 hypervariable regions of 16S rRNA on Illumina MiSeq platform (Illumina, Inc. Sand Diego, California) using forward and reverse primers. The forward primer set (28f-YM) consisted of a mixture of the following primers mixed at a 4:1:1:1 ratio; 28F-Borrellia GAGTTTGATCCTGGCTTAG; 28F- Chlorlex GAATTTGATCTTGGTTCAG; 28F- Bifido GGGTTCGATTCTGGCTCAG; 28F- YM GAGTTTGATCNTGGCTCAG. The reverse primer consisted of 388R GCTGCCTCCCGTAGGAGT. Sequencing was performed at Research and Testing Laboratories (RTL Genomics, Texas, USA).

Plants

Seed stocks	*Report on the source of all seed stocks or other plant material used. If applicable, state the seed stock centre and catalogue number. If plant specimens were collected from the field, describe the collection location, date and sampling procedures.*
Novel plant genotypes	*Describe the methods by which all novel plant genotypes were produced. This includes those generated by transgenic approaches, gene editing, chemical/radiation-based mutagenesis and hybridization. For transgenic lines, describe the transformation method, the number of independent lines analyzed and the generation upon which experiments were performed. For gene-edited lines, describe the editor used, the endogenous sequence targeted for editing, the targeting guide RNA sequence (if applicable) and how the editor was applied.*
Authentication	*Describe any authentication procedures for each seed stock used or novel genotype generated. Describe any experiments used to assess the effect of a mutation and, where applicable, how potential secondary effects (e.g. second site T-DNA insertions, mosiacism, off-target gene editing) were examined.*
